# Patterns of multimorbidity in association with falls among the middle-aged and older adults: results from the China Health and Retirement Longitudinal Study

**DOI:** 10.1186/s12889-022-14124-6

**Published:** 2022-09-24

**Authors:** Jingzheng Yan, Meijuan Wang, Yingjuan Cao

**Affiliations:** 1 School of Nursing and Rehabilitation, Cheeloo College of Medicine, Shandong University, NO. 107 Wenhua Xi Road, Jinan, China; 2 Department of Nursing, Qilu Hospital, Cheeloo College of Medicine, Shandong University, Jinan, China; 3 Nursing Theory & Practice Innovation Research Center, Shandong University, Jinan, China

**Keywords:** Multimorbidity patterns, Falls, Chronic diseases, CHARLS, China

## Abstract

**Background:**

Chronic diseases are important risk factors of falls. However, most studies explored the effect of a single chronic disease on falls and few studies explored the combined effect of multiple chronic diseases on falls. In this study, we examined the associations between falls and multimorbidity and multimorbidity patterns.

**Methods:**

Data collected between 2011 and 2018 were obtained from the China Health and Retirement Longitudinal Study (CHARLS). Multimorbidity was defined as the coexistence of ≥ 2 chronic diseases in the same person. The multimorbidity patterns were identified with exploratory factor analysis (EFA). The longitudinal associations of multimorbidity and multimorbidity patterns with falls were examined with generalized estimating equations methodology.

**Results:**

Compared with patients without chronic conditions, patients with one, two, and ≥ 3 chronic diseases had 37%, 85%, and 175% increased risk of falls, respectively. The EFA identified four multimorbidity patterns and the factor scores in the cardiac-metabolic pattern [adjusted odds ratio (aOR): 1.16, 95% confidence interval (95% CI): 1.12–1.20)], visceral-arthritic pattern (aOR: 1.31, 95% CI: 1.28–1.35), respiratory pattern (aOR: 1.12, 95% CI: 1.10–1.16), and mental-sensory pattern (aOR: 1.31, 95% CI: 1.28–1.35) were all associated with a higher risk of falls.

**Conclusion:**

Multimorbidity and multimorbidity patterns are related to falls. Older adults with multiple chronic diseases require early interventions to prevent falls.

## Introduction

As a common geriatric syndrome, falls are the leading cause of injury and death among the elderly. Approximately 50% of people aged > 80 years have experienced a fall [1]. Moreover, fall frequency increased with age and aggravated frailty [2]. In China, fall incidence has increased as the ageing population has increased rapidly in the past two decades [3]. Old people experiencing falls are more vulnerable to environmental challenges and face an increased risk of adverse outcomes and heavy medical burdens [4]. Therefore, identifying the potential risk factors of falls is of great importance [5].

Chronic diseases are important risk factors of falls in the elderly, but most studies focused on the independent effect of a single chronic disease on falls. Multimorbidity is defined as the co-occurrence of ≥ 2 chronic diseases, and preventing multimorbidity has become a priority in primary care [6]. Despite the large burden of multimorbidity in China, there has been little focus on the effect of multimorbidity on falls.

Previous explorations of the relationship between multimorbidity and falls rarely investigated the relationship between different multimorbidity patterns and falls [7, 8]. Multimorbidity patterns refer to the classification of chronic diseases into different combinations based on the associations between them [9, 10]. Chronic diseases belonging to the same pattern might interact with each other and lead to a further decline in physical performance and cognitive function [11]. Several studies have demonstrated inconsistent associations of different multimorbidity patterns with functional impairment and physical performance, which suggested that these phenomena might also exist between falls and different multimorbidity patterns [12–14]. However, there have been few investigations of the associations between multimorbidity patterns and falls in Chinese [15].

Accordingly, we determined the multimorbidity patterns in Chinese and the longitudinal associations between falls and multimorbidity and multimorbidity patterns based on a nationally representative cohort of middle-aged and old people in China. We expect that our findings will present medical workers and old people with more effective fall prevention suggestions.

## Materials and methods

### Study participants

Data were extracted from the China Health and Retirement Longitudinal Study (CHARLS). The CHARLS is a longitudinal cohort survey conducted by the Peking University National School of Development. From May 2011 to September 2011, 17,708 representative participants aged ≥ 45 years and their spouses were recruited to the CHARLS via multistage probability proportional to size sampling. The participants were from 150 counties and districts and 450 village-level units in China [16, 17]. In the CHARLS, demographic, socioeconomic status, and health status information was collected using questionnaire surveys and medical examinations. All participants underwent physical examinations and biochemical testing. After the baseline survey, the participants were followed-up every 2 years, during which similar baseline measurements were repeated. In this study, we used the baseline data collected in 2011 and the information collected in 2013, 2015, and 2018. After excluding participants who were lost to follow-up, a total of 10,015 participants were included in the final analyses.

### Definition of chronic diseases and multimorbidity

Data on the participants’ history of chronic diseases were collected with the following question: “Have you been diagnosed by a doctor as having the following chronic diseases (hypertension, dyslipidemia, diabetes, cancer, chronic lung diseases, liver diseases, heart disease, stroke, kidney diseases, memory-related diseases, digestive diseases, arthritis, and asthma)?” Depressive symptoms were assessed by the Center for Epidemiologic Studies of Depression Short Form (CES-D-10) [18] and participants with CES-D-10 scores ≥ 10 were defined as having depressive syndrome. Participants with emotional, neurological, or mental problems, or depressive syndrome were considered to have psychiatric diseases. Visual impairment and hearing loss were defined by self-reported poor vision and poor hearing, respectively. The number of chronic diseases was calculated as the sum of self-reported chronic diseases, psychiatric diseases, visual impairment, and hearing loss (range, 0–17). Multimorbidity was defined as the coexistence of ≥ 2 chronic diseases in the same person.

### Definition of falls

Information on falls was collected via a questionnaire survey. The participants were asked, “Have you fallen in the past 2 years?” The participants who answered “yes” were defined as having falls.

### Covariates

The covariates included age, sex, residence (rural or urban), marital status (married or cohabiting, or single), education level (illiterate, primary school or below, secondary school, high school or higher), smoking history, drinking history, physical activity level, and body mass index (BMI) (underweight, BMI < 18.5 kg/m^2^; normal weight, BMI = 18.5–24.9 kg/m^2^; overweight, BMI = 25–29.9 kg/m^2^; and obesity, BMI ≥ 30 kg/m^2^).

### Statistical analysis

The categorized data are presented as the frequency (percentage). Longitudinal associations between the number of chronic diseases and the presence of falls were explored using generalized estimating equation models.

The multimorbidity patterns were determined using exploratory factor analyses (EFA). The factors were extracted using the principal factor method based on tetrachoric correlation matrices. Factor interpretation was facilitated with an oblique rotation (Oblimin) of factor loading matrices. The data adequacy of our model was estimated using the Kaiser–Meyer–Olkin method and Bartlett test of sphericity. The number of factors identified was based on their interpretation, eigenvalue, and scree plot shape. For better robustness, chronic diseases with a prevalence < 3.0% were excluded, and those with a factor loading ≥ 0.40 were considered to be strongly associated. To obtain each participant’s factor score, the factor loading of each chronic disease was multiplied by 1 or 0 (presence or absence of chronic diseases, respectively), then each item was summed to calculate each participant’s total score (normalized to the mean value of 0 and standard deviation of 1).

The longitudinal associations between multimorbidity patterns and falls were examined with generalized estimating equation models. To assess the associations between different multimorbidity patterns and falls, the standardized factor score (mean = 0, standard deviation = 1) of each multimorbidity pattern and the number of chronic diseases in each pattern were included in the models. Then, the standardized factor scores were categorized into tertiles and the associations between each factor score tertile and falls were examined. For each generalized estimating equation model, the presence of falls was assumed to follow a binomial distribution. All statistical analyses were conducted using SPSS 25 (IBM). A two-sided *P* < 0.05 was considered statistically significant.

## Results

### Baseline characteristics of participants

Table 1 lists the participants’ baseline characteristics: 46.2% were male, 80.9% were aged < 65 years, 92.0% lived in rural areas, 86.1% had spouses, and 32.8% graduated from secondary school or higher. The overall prevalence of overweight and obese was 26.0% and 10.5%, respectively.

**Table 1:** Baseline characteristics of the participants (*n* = 10,015)

Characteristic	n (%)
Sex	
Male	4625 (46.2)
Female	5390 (53.8)
Age (years)	
45–65	8107 (80.9)
66 +	1908 (19.1)
Residence status	
Rural	9215 (92.0)
Urban	800(8.0)
Marital status	
Married and cohabiting	8625 (86.1)
Divorced/Separated/Widowed/Never married	1390 (13.9)
Level of education	
Illiterate	2548 (25.5)
Primary school or below	4175 (41.7)
Secondary school	2156 (21.5)
High school or above	1131 (11.3)
Smoking	
Yes	3034 (30.3)
No	6187 (61.8)
Quit	794 (7.9)
Drinking	
Drinks more than once a month	787 (7.9)
Drinks less than once a month	2530 (25.3)
Does not drink	6698 (66.9)
Physical check-up last year	
No	7422 (74.1)
Yes	2593 (25.9)
Number of chronic diseases	
None	1401(14.0)
1	2350(23.8)
≥ 2 (multimorbidity)	6229(62.2)
BMI	
Underweight	489 (4.9)
Normal	4624 (46.2)
Overweight	2601 (26.0)
Obese	1053 (10.5)
Falls	
Yes	1523 (15.2)
No	8487 (84.7)

### Association between the number of chronic diseases and falls

An increased number of chronic diseases was associated with an increased risk of falls (Table 2). After multivariable adjustment, the risk of falls increased by 37% in participants with one chronic disease, 80% in participants with two chronic diseases, and 175% in participants with ≥ 3 chronic diseases as compared with the participants without chronic diseases.

**Table 2:** Associations between the number of chronic diseases and falls among middle-aged and aged people in China (*n* = 10,015)

Number of chronic diseases	Crude		Adjusted	
	OR (95% CI)	*P*	OR (95% CI)	*P*
0	Ref	**/**	Ref	**/**
1	1.40 (1.24,1.58)	0.001	1.37 (1.22,1.55)	0.001
2	1.87 (1.66,2.11)	0.001	1.80 (1.60,2.03)	0.001
≥ 3	2.99 (2.67,3.34)	0.001	2.75 (2.46,3.08)	0.001

Adjusted for age, sex, marital status, education level, household income per capita, residential region, smoking status, drinking status, and body mass index

*OR* odds ratio, *CI* confidence interval

### Multimorbidity patterns

A total of 14 chronic diseases with a prevalence of ≥ 3% at baseline were included in the factor analysis (Table 3). Four multimorbidity patterns were identified: cardio-metabolic (hypertension, dyslipidemia, diabetes, heart problems, stroke), visceral-arthritic (liver diseases, kidney diseases, digestive diseases, arthritis), respiratory (chronic lung diseases and asthma), and mental-sensory (psychiatric conditions, vision impairment, hearing loss).

**Table 3:** Factor loadings of the multimorbidity patterns for each disease

Chronic diseases	Factor^a^			
	Cardio-metabolic pattern	Visceral-arthritic pattern	Respiratory pattern	Mental-Sensory pattern
Dyslipidaemia	0.69	0.13	-0.01	-0.07
Diabetes	0.61	0.02	0.00	-0.03
Hypertension	0.61	-0.09	0.04	0.06
Cardiac diseases	0.47	0.29	0.17	0.04
Stroke	0.43	0.03	-0.01	0.11
Digestive diseases	-0.06	0.65	0.04	0.07
Arthritis	0.00	0.55	0.06	0.23
Kidney diseases	0.13	0.53	0.04	-0.03
Liver diseases	0.09	0.48	0.03	-0.11
Asthma	0.04	0.02	0.83	0.03
Chronic lung diseases	0.04	0.14	0.81	0.04
Visual impairment	0.04	-0.01	-0.03	0.74
Hearing loss	0.06	-0.01	0.09	0.69
Psychiatric diseases	0.01	0.35	0.00	0.40

^a^Note: Kaiser–Meyer–Olkin value is 0.68; Bartlett’s test of sphericity: *P* < 0.001

### Longitudinal associations between multimorbidity patterns and falls

Table 4 displays the longitudinal associations between the multimorbidity patterns and falls. After the adjustment, increased factor scores and increased number of chronic diseases of the cardio-metabolic, visceral-digestive-arthritic, respiratory, and mental-sensory patterns were associated with a higher risk of falls. Moreover, compared with participants with factor scores in tertile 1 (T1) of each pattern (except the respiratory pattern), participants with factor scores in T3 had a higher risk of falls, with odds ratios (ORs) of 1.21 (95% confidence interval [95% CI]: 1.14, 1.29) to 1.84 (95% CI: 1.72, 1.97).

**Table 4:** Associations between falls and multimorbidity patterns among middle-aged and aged people in China (*n* = 10,015)

Variable (reference)	Cardio–metabolic pattern		Visceral-arthritic pattern		Respiratory pattern		Mental-sensory pattern	
	OR (95% CI)	*P*	OR (95% CI)	*P*	OR (95% CI)	*P*	OR (95% CI)	*P*
Factor score^a^	1.16 (1.12,1.20)	< 0.01	1.31 (1.28,1.35)	< 0.01	1.12 (1.10,1.16)	< 0.01	1.31 (1.28,1.35)	< 0.01
Number of diseases	1.15 (1.12,1.19)	< 0.01	1.37 (1.33,1.42)	< 0.01	1.31 (1.22,1.39)	< 0.01	1.45 (1.41,1.50)	< 0.01
Tertile of factor score								
T1	Ref	**/**	Ref	**/**	Ref	**/**	Ref	**/**
T2	1.33 (1.16,1.52)	< 0.01	1.49 (1.38,1.61)	< 0.01	1.43 (1.31,1.58)	< 0.01	1.69 (1.58,1.81)	< 0.01
T3	1.21 (1.14,1.29)	< 0.01	1.76 (1.64,1.89)	< 0.01	1.48 (1.28,1.70)	< 0.01	1.84 (1.72,1.97)	< 0.01

Note: Adjusted for age, sex, marital status, education level, household income per capita, residential region, smoking status, drinking status, body mass index, and follow-up duration

The number of diseases in each pattern was considered a continuous variable

*OR* adjusted odds ratio, *CI* confidence interval

^a^The factor score for each multimorbidity pattern was standardized to a mean of 0 and a standard deviation of 1 and was used as a continuous variable

## Discussion

In the present study, we identified four multimorbidity patterns (cardiac-metabolic, visceral-arthritic, respiratory, and mental-sensory) in a nationally representative sample of community-dwelling middle-aged and old Chinese. We determined that multimorbidity and each multimorbidity pattern were positively associated with an increased risk of falls.

In this study, participants with ≥ 2 chronic diseases were more likely to fall than those without chronic diseases, which was consistent with the results of Lawlor et al*.* and Immonen et al*.* [19, 20]. Immonen et al*.* [20] reported higher incident and relapse rates of falls as the number of chronic diseases increased, which indicated that chronic diseases might exert cumulative effects on the occurrence of falls. Frailty might explain the relationship between multiple diseases and falls [21, 22], where people with multiple diseases have accelerated catabolism and are more likely to be frail, which could increase the risk of falls. Polypharmacy is another possible explanation for these associations in people with multimorbidity. Polypharmacy was significantly related to falls, and the effect of drug interactions was more obvious in the elderly due to degradation of the drug absorption, metabolism, and elimination processes [23, 24]. Zia et al. reported that taking ≥ 4 drugs increased the incident rate of falls [25]. Our findings suggested that people with multiple chronic diseases should adopt effective interventions for preventing falls. Furthermore, medical workers should focus more on such patients.

Different studies reported differing numbers of multimorbidity patterns. A systematic review reported that the Western population had three common multimorbidity patterns: metabolic diseases, mental health problems, and musculoskeletal diseases [10]. Another systematic review of multimorbidity patterns in Asian populations revealed that Asians exhibited five common comorbidity patterns: cardiovascular and metabolic diseases, mental health problems, degenerative diseases, pulmonary diseases, and cancer [26]. In the present study, we identified four multimorbidity patterns. The reasons for these inconsistencies might be complicated. First, due to regional and ethnic differences, the prevalence of chronic diseases differs between study populations. Second, differing chronic diseases were included for determining the multimorbidity pattern. Third, different studies did not use consistent statistical analysis methods to determine the multimorbidity patterns.

The cardiac-metabolic pattern was the most common pattern in both Asian and Western populations [26, 27]. Diseases in the cardiac-metabolic pattern share common risk factors and can prompt each other mutually. The chronic diseases included in the cardiac-metabolic pattern, specifically hypertension, diabetes, and heart disease, could all increase the risk of falls [28–30]. Patients with hemodynamic abnormalities were more likely to experience dizziness, which might lead to unconscious falls [31].

The diseases included in the respiratory pattern, such as chronic obstructive pulmonary disease (COPD) and pneumonia, can cause complications such as hypoxia, anemia, dehydration and electrolyte disorders, which can weaken a person’s compensatory capacity and balance ability and thereby increase the risk of falls [32]. Many studies demonstrated that chronic respiratory diseases, such as COPD and asthma, are closely related to falls [15, 22, 32, 33], and patients with multiple chronic respiratory diseases (respiratory pattern) have a higher risk of falls.

Our findings demonstrate that the effects of the number of chronic diseases and the factor scores within different multimorbidity patterns on falls were inconsistent and that the increased risk of falls was higher in the visceral-arthritic and mental-sensory patterns. This finding suggests that people who have more diseases within these two multimorbidity patterns face a higher risk of falls. There are several reasons for this phenomenon: in the visceral-digestive-arthritis pattern, pain, deformity, and dynapenia caused by musculoskeletal diseases, such as arthritis, further reduced patients’ motor abilities; therefore, these patients were more likely to fall [34, 35]. Furthermore, chronic kidney diseases prompted the occurrence of osteoporosis, which is highly related to falls and fractures in the elderly [35].

In the mental-sensory pattern, hearing loss and visual impairment make it difficult for patients to avoid obstacles and potential dangers when moving, so the patients are more likely to fall. Moreover, mental disorders, such as depression and anxiety, were highly correlated with falls [36, 37]. Fatigue and lack of motivation also led to a decline in functional ability, muscle strength, and balance ability, which might exert a cumulative impact on falls. Moreover, due to difficulties in the early identification of mental and sensory disorders, the diagnosis of such disorders in the elderly is likely to be delayed, thereby increasing the incidence of falls [36].

As our study population was from a rural or community setting, the findings may be able to provide some insight into primary care. The primary prevention of falls in the elderly should emphasize patients with multimorbidity, especially those with a high number of chronic conditions. Older adults with different multimorbidity patterns should be offered the appropriate fall prevention management measures focusing on the multimorbidity patterns with a higher risk of falls, such as the visceral-arthritis and mental-sensory patterns in this study. Early prevention should be implemented and a multidisciplinary fall prevention team should tailor fall prevention management plans for such patients. Communication between health practitioners and older adults with multimorbidity is also important, with a focus on safety education and awareness of fall prevention. Fall prevention research aimed at optimal and immediate transferability to real-world clinical practice is imperative.

The present study has several strengths. To the best of our knowledge, this is the first study to investigate the longitudinal association between different multimorbidity patterns and falls among Chinese. Second, the CHARLS collected data over a long duration from a nationally representative cohort of middle-aged and aged people in China, including detailed information on falls and most chronic diseases. Third, the EFA is the preferred method for exploring multimorbidity patterns.

Our study also has several limitations. First, the chronic diseases were self-reported and there might have been recall bias. Second, participants with missing data on chronic diseases were considered to have no chronic disease, which might have resulted in slight deviations in the multimorbidity prevalence and factor scores. Third, detailed information on the disease severity was not included in this study due to data availability.

## Conclusion

The number of chronic diseases was positively associated with an increased risk of falls. Four multimorbidity patterns were identified in Chinese, which all increased the risk of falls. Early interventions are recommended for people with multiple chronic diseases to prevent falls. Future research is needed to elucidate the mechanism of the relationship between falls and multimorbidity and the multimorbidity pattern.

## Data Availability

The data of this study are available at [http://charls.pku.edu.cn/index/en.html]. [The CHARLS] Yaohui Zhao, et al.; 2018; Harmonized CHARLS; the Gateway to Global Aging Data; Version C; http://charls.pku.edu.cn/pages/data/harmonized_charls/en.html.
